# Evolving factors influencing consumers’ attitudes towards the use of eHealth applications: implications on the future of Neom

**DOI:** 10.1093/inthealth/ihab020

**Published:** 2021-05-26

**Authors:** Areej Algumzi

**Affiliations:** College of Business Administration, University of Tabuk, Saud Arabia

**Keywords:** attitudes, digital health, eHealth, influencing factors

## Abstract

**Background:**

Factors affecting the adoption and use of eHealth applications have been extensively researched from different perspectives in different regions. However, with the changing circumstances (e.g. the coronavirus disease 2019 pandemic), new influencing factors might evolve and can influence the attitudes of consumers towards using eHealth applications. The purpose of this study was to identify and evaluate the evolving factors affecting consumer attitudes towards the use of eHealth applications and provide implications for the future of Neom.

**Methods:**

An online survey questionnaire was used to collect data from 976 eHealth consumers in Saudi Arabia, which included 527 male and 449 female participants. Findings were analysed using the statistical means and standard deviations for each item in the questionnaire to analyse the role of each factor in depth. Statistical t-tests were used to identify significant differences between the groups categorised by age and gender.

**Results:**

‘Necessity but not interest’ (mean 4.5 [standard deviation {SD} 1.12]) and fear (mean 4.5 [SD 1.13]) and psychological factors including depression (mean 4.4 [SD 1.54]), stress (mean 4.2 [SD 1.09]) and anxiety (mean 4.3 [SD 1.61]) were identified to be major evolving influencing factors, while other factors including performance expectancy, ease of use, enjoyment and incentives were identified to be comparatively less influential.

**Conclusions:**

Increasing adoption of eHealth mainly due to necessity but not out of interest can have serious implications for patients and the adoption of eHealth technologies in the future.

## Introduction

Healthcare is one of the most important sectors that needs to be effectively managed in order to protect human capital and enhance national development. Globally there are only 10 medical doctors available per 10 000 population and >26% of the global population has <3 doctors per 10 000 population, reflecting the huge scarcity in the healthcare workforce. However, countries with a lower relative need have the highest number in the healthcare workforce, while countries with the highest relative need have the lowest number, especially in the African region, which suffers with >22% of the global burden of disease and has access to only 3% of the healthcare workforce.^1^ As a result, the average global health index score stood at 40.2 out of 100, reflecting the poor international preparedness for healthcare needs and challenges such as epidemics and pandemics.^2^ Although countries invest local and donor funds in healthcare, very few countries are investing in activities such as health security gaps assessment and preparing of action plans.^2^ The USA was the highest contributor to healthcare services across the world, investing 17.9% of its gross domestic product on healthcare services in 2019.^3^ However, developing and underdeveloped countries that are already suffering from various internal and external issues may not be able to create sufficient funding for healthcare. Considering these factors, the need for health interventions such as eHealth has been increasing. According to a recent report on the global healthcare outlook,^4^ the need for predictive and preventive care; cheaper, precise and less invasive treatments; and balancing user demands were identified to be the main drivers for digital health interventions. In addition, developments in innovative technologies such as artificial intelligence (AI), blockchain systems, cloud computing, robotics, natural language processing (NLP), machine learning (ML), sensors and wearable technologies are driving eHealth solutions; however, cybersecurity remains one of the greatest challenges.^4^ But improving eHealth interventions comes at great costs. The total global eHealth industry funding in 2019 was reported as $13.7 billion, which is projected to reach $38 billion by 2025.^5^

Focusing on the amount of spending, an increase in healthcare investments was observed across many nations. For instance, healthcare spending in Brazil, Russia, India, China and South Africa (BRICS) is growing and constitutes an important part of governmental efforts to address population need and healthcare systems.^6,7^ There is a need to increase healthcare spending in order to address the growing healthcare needs of people in different countries. To achieve effective outcomes, investments in cyber physical systems, AI technology and accelerated innovation in the field of eHealth will change the workflow in medical care and inevitably transform the labour market in upcoming decades.^8^ These technologies promote the increased use of eHealth by improving services with cost-effective investments and efficient resource allocation and utilization. However, with countries such as Saudi Arabia providing free access to healthcare, an additional burden is placed on the government that can affect service delivery.^9^ According to the Global Health Index,^10^ Saudi Arabia ranks 89th in prevention of diseases, 114th in responding to healthcare needs, 81st in health norms and compliances and 71st in the severity of risks in healthcare among 195 countries, reflecting its unpreparedness for handling healthcare challenges. However, the country ranks 35th among the 195 countries in healthcare infrastructure and equipment, revealing access to advanced healthcare diagnosis and treatment equipment.^10^ As a part of its National Transformation Program to decrease reliance on an oil-based economy and move towards a knowledge-based economy by initiatives such as Saudization, various healthcare initiatives are being undertaken. A rapid increase in expenditures, reduction in waiting times, addressing issues such as a shortage of healthcare resources and digitization of healthcare operations and services are a few objectives of the development program.^11,12^

Supported by adoption of innovative technologies, Saudi Arabia as a part of its Vision 2030 programme is building a city called Neom on the coast of the Red Sea, with an initial investment of $500 billion. The city is being planned to accommodate more than 1 million international and local residents and act as a hub for entrepreneurs, innovators and research.^13–1^^7^ Various eHealth plans were proposed for development of the healthcare infrastructure within the city, connected through high-end technology based on AI and predictive technologies.^18,19^ However, it is unclear if the Saudi population is ready to experience such a rapid transformation in accessing healthcare services. Most of the studies^20–22^ identified factors in the context of usability, eHealth awareness and behavioural factors in using eHealth applications. However, there is a lack of research on eHealth in Saudi Arabia, especially on the use of eHealth research^23,24^ during the transformation process through Vision 2030 initiatives. In addition, the coronavirus disease 2019 (COVID-19) pandemic has affected patients’ attitudes towards the use of eHealth applications, as the pandemic has greatly influenced socio-economic conditions. However, various organizational and technical measures were necessary in order to promote the effective use of healthcare systems during the pandemic.^25^ Therefore there is a need to identify the evolving factors influencing the use of eHealth technologies in Saudi Arabia in order to assess the preparedness of the Saudi people to experience advanced and predictive healthcare technologies in the near future, especially in Neom. Accordingly, the purpose of this study was to identify and evaluate the evolving factors affecting consumer attitudes towards the use of eHealth applications and the implications for the future of Neom.

## Literature review

Attitude-based studies^26–30^ on eHealth have mainly focused on accessing online health information, sharing online health information and the factors influencing the adoption of online health applications. These attitudes are influenced by various other factors. For example, the online information-seeking behaviour of patients was correlated with healthcare professional's ability to support them and their adherence to medical prescriptions.^26^ Limited doctor consultation time and barriers to accessing professional health services were other factors identified that influence online health information-seeking behaviour. Thus it was determined that older and less-educated individuals are among those who seek online health information.^27^ However, this may not be the case in all regions, as factors such as internet skills and socio-economic aspects may cause individuals to seek private healthcare access. Accordingly, factors such as age, education, income, eHealth literacy, location (rural and urban areas), perceived health and social isolation were identified as influencing the approach towards accessing online health information.^28–30^

In addition, knowledge-sharing behaviours on online platforms may influence user's attitudes towards eHealth applications. In a comparative study^31,32^ of healthcare professionals and normal users it was determined that reciprocity and altruism positively affect the knowledge-sharing intention of both health professionals and normal users. In addition, reputation and knowledge self-efficacy have a greater influence on the knowledge-sharing intentions of health professionals compared with normal users, whereas reciprocity, altruism and empathy have a greater influence on the knowledge-sharing intentions of normal users compared with health professionals. Therefore attitudes of professionals and normal users may influence the use and adoption of eHealth applications in different ways. For example, in another comparative study^33^ on professionals and cancer patients, it was identified that while professionals reflected fear over giving online access to their medical records for patients, while patients did not have any anxiety or concerns, but were well-prepared for the course of treatment. While studies identified that online knowledge sharing can create a sense of togetherness and support among patients and increase their awareness and experiences about their disease, complication and treatments, there are issues such as the credibility and reliability of online information^34^ and the limitation that not all types of patients^35^ can access and share online health information.

Various other factors have been identified^36^ that can influence the adoption and continuous use of eHealth applications, including the following:

- Performance expectancy: defined as the degree to which an eHealth application would enhance the productivity of its users.^37^ For example, if patients are more attracted to a certain eHealth application, they may seek more information and knowledge from that application and increase their awareness levels. In this context, the productivity of users is analysed in terms of creating awareness.
- Ease of use: also called effort expectancy, which is the ‘ease’ associated with an eHealth application.^38^ An eHealth application may be adopted effectively by large number of users if they are able to use it easily.
- Social influence: the extent to which an eHealth application's use and adoption is influenced by the people in society. Consumers’ decisions are shaped by the influence of social factors, including word of mouth, friends, family and others who are a part of their social network.^39^
- Enjoyment: reflects the fun part, as perceived by its users, in the use of an eHealth application. If the users enjoy a technology, there is a good chance that they will want to use it again.^40^
- Incentives: these are promotions such as discounts or coupons offered for using eHealth applications, which may boost the adoption of an application.^41^
- Facilitating conditions: reflects the conditions that support the use of eHealth applications.^42^ For example, good internet and communication skills and eHealth awareness allow users to use eHealth applications more effectively.
- Aesthetics: the feature of an eHealth application relating to the design, animation or visual elements that capture the user's attention.^43^
- Trust: the reliability and credulity of an eHealth application. It can also be understood as the extent to which a consumer has faith in an eHealth application.^36^
- Satisfaction: the response of consumers to eHealth applications.^36^ Customer satisfaction was found to be a major determinant of continuous intention in a number of mobile technologies and applications.^44^

In addition, there are other factors related to the current situation arising out of the COVID-19 pandemic. Studies^45,46^ have focused on the fear and stress factors relating to healthcare workers in treating COVID-19 patients. However, fear about contamination and being affected by COVID-19 or fear about an inability to access healthcare can also influence normal users’ adoption of eHealth applications. Other psychological factors in a similar context, including stress, anxiety and depression,^47^ can also influence the adoption of eHealth. In addition, necessity is another important factor. During the lockdowns and curfews there was limited access to outpatient healthcare and it was necessary for people to rely on eHealth applications to address their healthcare needs. As a result, it was necessity rather than interest that may have dominated the adoption of eHealth during the pandemic. This may affect future adoption, as patients may move back towards traditional outpatient treatment or they may continue to use eHealth options.

## Methods

### Study design

**Figure 1.**
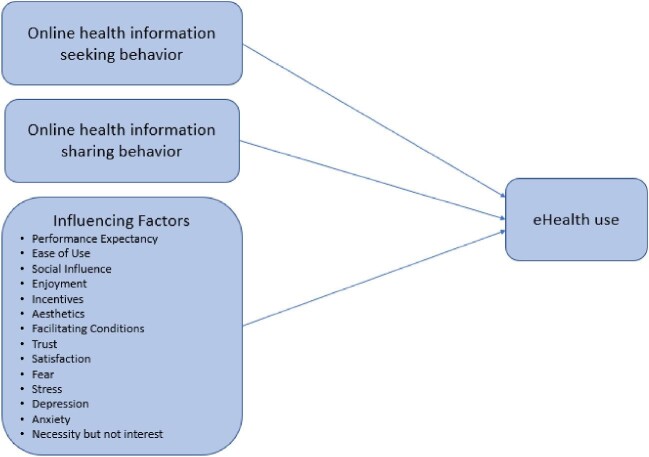
Study design.

Studies identified in the literature review found various attitudes of eHealth consumers relating to online health information-seeking behaviour,^25–29^ online health information-sharing behaviour^31–35^ and other factors^36–46^ that could influence the use of eHealth applications. Few new factors in the current COVID-19 context, such as stress, fear, anxiety, depression and necessity, were identified, which may influence the use of eHealth in future. An online survey questionnaire was adopted to evaluate the impact of identified factors on the use of eHealth applications in Saudi Arabia. Accordingly, part A of the eHealth impact questionnaire^48^ was adopted in this study for assessing attitudes towards online health information and sharing health experiences on online platforms. In addition, various factors of influence, which included 14 items, were identified from the literature review^36–47^ and were included in the questionnaire to evaluate their impact on the use of eHealth use, as shown in Figure 1. These 14 items are grouped into three categories: application features (aesthetics, ease of use, incentives), external factors (social influence, facilitating conditions) and behavioural factors (performance expectancy, trust, satisfaction, enjoyment, fear, stress, depression, anxiety, necessity but not interest).

The survey questionnaire was designed in two parts. The first part provides an introduction to the survey, a brief description of the purpose of study, data usage policy, privacy aspects of the study and study objectives. At the end of first section, an acceptance button is provided where participants provide consent. The second part of the questionnaire included items in three categories: online health information-seeking behaviour, online health information-sharing behaviour and other factors of influence. Items related to each category in the questionnaire were rated using a 5-point Likert scale.^49^ The questionnaire was then translated into Arabic by two professional Arabic translators.

A pilot study was conducted with 12 randomly selected individuals on online health portals. Cronbach's α (0.70–0.81)^50^ was used for calculating the reliability of the questionnaire items, indicating good reliability and consistency. In addition, feedback was collected from all the participants in the pilot study. Based on this feedback, a few words were rewritten in Arabic to reflect their meaning more accurately in relation to the items in the English version of the questionnaire. The Arabic version of the questionnaire was then uploaded to the QuestionPro application,^51^ generating a link to the questionnaire.

### Recruitment and sampling

As the objective of this study was to identify and evaluate the evolving factors affecting the attitudes of consumers using eHealth applications, the need to include a diverse group with a large sample was realised. Accordingly, the survey link was forwarded to various online health groups and communities and also to other groups on various social platforms using a wide range of applications, including WhatsApp, Facebook, Instagram and Twitter. In addition, a snowball sampling^52^ technique was used to increase the sample population by inserting a request in the message attached with the survey asking the participants to forward the message and survey link to their peers, friends and family members. The survey was conducted over a period of 6 weeks, from 8 October 2020 to 19 November 2020. The survey link was initially forwarded to 869 participants using the various methods described above. As a result of using snowball sampling, a total of 1141 responses were received. Of the 1141 respondents, 165 partly completed the survey, thus a final sample of 976 was achieved, reflecting a response rate of 85.5%.

### Data analysis

The responses for the questionnaire items were downloaded from the QuestionPro application into an Excel spreadsheet (Microsoft, Redmond, WA, USA). Average ratings (means) and standard deviations (SDs) for each item were calculated in order to prioritise the opinions of the participants and to analyse the variance in responses.

## Results

The final sample achieved for the study was 976 (Table 1). Survey participants were almost equally distributed across genders (54% males and 46% females). Focusing on the distribution of participants across age groups, the majority of the participants were between 20 and 39 y of age, including 28.9% in the 20–29 y age group and 31.4% in the 30–39 y age group. Among the remaining participants, 22.6% were in the 40–49 y age group, followed by 12.9% in the 50–59 y age group and 4.2% who were >59 y of age. Almost 65% of the participants were educated, including 31.9% with a bachelor's degree, 16.9% with a master's degree, 14.9% with other education and 2.8% with a doctoral degree. Participants were almost equally distributed across all the administrative regions: 26.9% from the central region, 21% from the western region, 20.3% from the eastern region, 19.9% from the northern region and 11.9% from the southern region.

**Table 1.** Frequency distribution of demographic variables.

Variables	n (%)
Gender	
Male	527 (54)
Female	449 (46)
Age (years)	
20–29	283 (28.9)
30–39	306 (31.4)
40–49	221 (22.6)
50–59	125 (12.9)
>59	41 (4.2)
Education	
Bachelor's degree	311 (31.9)
Master's degree	165 (16.9)
PhD	27 (2.8)
Other	146 (14.9)
Uneducated	327 (33.5)
Region	
North (Jawf, northern borders)	194 (19.9)
West (Tabuk, Medina, Mecca, Al Bahah)	205 (21)
Central (Ha'il, Qasim, Riyadh)	263 (26.9)
East (Eastern Province)	198 (20.3)
South (Asir, Najran, Jazan)	116 (11.9)

The information-seeking behaviour on online platforms was correlated with the support and advice received from healthcare professionals and their adherence to medical prescriptions.^26^ Accordingly, the findings in this study reflected that the participants used the internet to crosscheck the advice given by their doctors (mean 3.9 [SD 4.29]). However, there was a difference of opinion observed in this context, as the variance (SD 4.29) was high, reflecting the responses away from the mean. It was also observed that few participants relied on the internet to understand what their doctor said (mean 3.3 [SD 1.65]). In addition, there were a considerable number of participants who used the internet to analyse their symptoms and decide if they needed to visit the doctor. Overall, reliance on the internet was identified to be medium to high in seeking health-related information, as shown in Table 2.

**Table 2.** Attitudes towards online health information

Items	Mean	SD
The internet is a reliable resource to help me understand what a doctor tells me.	3.3	1.65
The internet can help the public to know what it is like to live with a health problem.	2.8	1.31
The internet can be useful to help people decide if their symptoms are important enough to go to see a doctor.	2.9	1.28
I would use the internet if I needed help to make a decision about my health (for example, whether I should see a doctor, take medication or seek other types of treatment).	3.1	1.41
I would use the internet to check that the doctor is giving me appropriate advice.	3.9	4.29

To further analyse the results, the differences in the attitudes between male and female participants were analysed (Table 3). Significant differences between male (mean 2.5 [SD 1.86]) and female (mean 3.9 [SD 2.11]) participants were identified in relation to the attitudes towards online health information, with t=11.0155 and p<0.0001 (confidence interval [CI] 0.05). Differences were observed in relation to the attitude to crosscheck a doctor's advice and using online information to understand a doctor's advice.

**Table 3.** Difference in attitudes towards online health information (by gender)

Gender	N	Mean	SD	df	t-Value	p-Value
Male	527	2.5	1.86	974	11.0155	<0.0001^*^
Female	449	3.9	2.11			

df: degrees of freedom.

* Significant at p=0.05.

Further analysis of results by age groups is presented in Table 4. Significant differences between participants ≤39 y of age (mean 2.9 [SD 1.43]) and participants >39 y of age (mean 3.9 [SD 2.11]) were identified in relation to the attitudes towards online health information (t=11.0155, p<0.0001 [CI 0.05]). Differences were observed in relation to the attitude to crosscheck a doctor's advice and to check online information to analyse symptoms and decide whether or not to visit the doctor.

**Table 4.** Difference in attitudes towards online health information (by age)

Age (years)	N	Mean	SD	df	t-Value	p-Value
≤39	589	2.9	1.43	974	4.7092	<0.0001^*^
>39	387	3.5	2.54			

df: degrees of freedom.

* Significant at p=0.05.

The findings identified were similar to those in Graffigna et al.,^26^ which analysed an older population that reflected a positive attitude towards online health information-seeking behaviour and supported findings^22–29^ that age and gender influence information-seeking behaviour.

The participants’ attitude towards sharing their health experiences online are presented in Table 5. Reassuring themselves that there are other people with the same health complications (mean 4.1 [SD 1.32]) and real-time access to information (mean 3.9 [SD 1.84]) reflected their attitudes relating to togetherness and accessibility, respectively. In addition, the idea of using the internet to share if they were unable to share health issues with family or peers (mean 3.6 [SD 1.79]) reflected that the majority of the participants feel free to share with others online. These findings were similar to those reflected by others^33,34^ towards sharing information.

**Table 5.** Attitudes towards sharing health experiences online

Items	Mean	SD
The internet is a good way of finding other people who are experiencing similar health problems.	3.2	1.14
It can be helpful to see other people's health-related experiences on the internet.	3.4	1.26
The internet is useful if you don't want to tell people around you (for example, your family or people at work) how you feel.	3.6	1.79
It can be reassuring to know that I can access health-related websites at any time of the day or night.	3.9	1.84
The internet is a good way of finding other people who are facing health-related decisions I may also face.	3.3	1.51
Looking at health-related websites reassures me that I am not alone with my health concerns.	4.1	1.32

The results were further analysed to see if there were differences in the attitudes towards sharing health experiences between genders (Table 6). Significant differences between male (mean 3.9 [SD 1.75]) and female (mean 3.2 [SD 1.20]) participants were identified in relation to attitudes towards sharing health experiences (t=7.1616, p<0.0001 [CI 0.05]). Differences were observed in relation to the attitude of sharing online rather than with family (observed among females) and real-time access to health-related websites (observed among males).

**Table 6.** Difference in attitudes towards sharing health experiences online (by gender)

Gender	N	Mean	SD	df	t-Value	p-Value
Male	527	3.9	1.75	974	7.1616	<0.0001^*^
Female	449	3.2	1.20			

df: degrees of freedom.

* Significant at p=0.05.

Further analysis of results by age groups is presented in Table 7. Significant differences between participants ≤39 y of age (mean 4.1 [SD 1.41]) and participants >39 y of age (mean 3.1 [SD 1.54]) were identified in relation to the attitudes towards sharing health experiences online (t=10.4466, p<0.0001 [CI 0.05]).

**Table 7.** Difference in attitudes towards sharing health experiences online (by age)

Age (years)	N	Mean	SD	df	t-Value	p-Value
≤39	589	4.1	1.41	974	10.4466	<0.0001^*^
>39	387	3.1	1.54			

df: degrees of freedom.

* Significant at p=0.05.

Differences were observed in relation to the attitudes relating to anytime accessibility (by age >39 y) and sharing online rather than sharing with family (by age ≤39 y). The differences in genders and ages were evident supporting the findings in other studies.^33,34,53,54^

Almost all the factors identified in the study (Table 8) were found to highly influence the participants in using eHealth applications except incentives (mean 3.1 [SD 3.42]). However, opinions among the participants varied widely in relation to the incentives factor, indicating that few participants were highly influenced by incentives while others were not. ‘Necessity but not interest’ (mean 4.5 [SD 1.12]) and fear (mean 4.5 [SD 1.13]) were identified to be the most significant influential factors, reflecting that the majority of the participants were using eHealth arising out of necessity and fear. With huge investments being made in eHealth in Saudi Arabia, similar to BRICS,^6,7^ for improving cost-effectiveness, efficient resource allocation^8^ and improved service deliver, the current attitude ‘necessity but not interest’ can be a cause for concern, as it may affect the use of eHealth systems in the future. Thus there is a need to create awareness among the citizens about the benefits of eHealth and address any concerns they have such as privacy and security. In addition, depression (mean 4.4 [SD 1.54]), stress (mean 4.2 [SD 1.09]) and anxiety (mean 4.3 [SD 1.61]) were identified to be other psychological factors influencing the use of eHealth applications. As identified in other research,^45–47^ increasing anxiety, stress and depression; an inability to access in-person healthcare services; and fear of contamination in hospitals might have made eHealth applications a necessity. Factors including ease of use, performance expectancy, enjoyment, aesthetics and facilitating conditions were influencing factors in the use of eHealth, three important factors—social influence, trust and satisfaction—reflected very high influence in using eHealth applications.

**Table 8.** Factors influencing the use of eHealth applications

Category	Items	Mean	SD
App features	Aesthetics	3.8	4.17
	Ease of Use	4.2	1.82
	Incentives	3.1	3.42
Behavioural factors	Enjoyment	3.7	1.11
	Performance expectancy	3.8	1.03
	Necessity but not interest	4.5	1.12
	Anxiety	4.3	1.61
	Trust	4.4	1.29
	Satisfaction	4.3	1.07
	Fear	4.5	1.13
	Stress	4.2	1.09
	Depression	4.4	1.54
External factors	Social influence	4.4	1.41
	Facilitating conditions	3.8	2.35

The results were further analysed to see if there were differences in the influencing factors perceived by the participants among genders (Table 9). Significant differences between male (mean 3.8 [SD 1.87]) and female (mean 4.3 [SD 1.59]) participants were identified in relation to attitudes towards influencing factors (t=4.4569, p<0.0001 [CI 0.05]).

**Table 9.** Difference in opinions towards influencing factors (by gender)

Gender	N	Mean	SD	df	t-Value	p-Value
Male	527	3.8	1.87	974	4.4569	<0.0001^*^
Female	449	4.3	1.59			

df: degrees of freedom.

* Significant at p=0.05.

Behavioural factors were identified as influencing the use of eHealth applications (mean 4.2) more than external factors (mean 4.1) and application features (mean 3.7). Differences were observed in relation to the ease of use, enjoyment, trust, social influence, fear, anxiety, stress (observed among females) and necessity, depression, performance expectancy and facilitating conditions (observed among males).

Further analysis of results by age groups is presented in Table 10. No significant differences between the participants ≤39 y of age (mean 4.2 [SD 1.37]) and participants >39 y of age (mean 3.9 [SD 2.09]) were identified in relation to the attitudes towards influencing factors (t=0.0431, p=0.96 [CI 0.05]).

**Table 10.** Difference in opinions towards influencing factors (by age)

Age (years)	N	Mean	SD	df	t-Value	p-Value
≤39	589	4.2	1.37	974	0.0431	0.9657
>39	387	3.9	2.09			

df: degrees of freedom.

Differences were observed in relation to fear, trust, satisfaction, stress, anxiety (by age >39 y) and enjoyment, aesthetics, performance expectancy and ease of use (by age ≤39 y).

Thus all the factors identified in prior research^36–47^ were found to have medium to very high impact on consumers using eHealth applications.

## Conclusions

The diverse sample population included in this study, distributed across gender, age, education and location, represented proportionate levels for effective analysis of data. Regarding the attitudes of the participants relating to online health information, most of the participants saw it as an additional source to gain clarity, increase understanding and raise awareness about their healthcare issues and indicated it was a good approach for information-seeking behaviour. Females and older participants reflected a greater preference towards online health information seeking, reflecting a lack of knowledge and awareness as the main drivers for information seeking. However, in relation to sharing health experiences online, male and younger participants (≤39 y) indicated a greater preference compared with females and older participants, indicating high levels of online interactivity and social sharing behaviour among males and younger participants. Thus the information-seeking behaviour and sharing behaviour reflects the preparedness of the participants in the national transformation process of digitizing healthcare services with high-end AI and predictive technologies in Neom. In relation to the factors of influence, this study contributes an important finding that psychological factors such as fear, stress, anxiety and depression can greatly influence the use of eHealth applications. In addition, the influence of the necessity factor reflects that participants are being forced to accept the change in shifting from traditional healthcare services to online healthcare services. This is an interesting finding, as it can have serious implications on their health and the use of eHealth applications in the future. Other factors such as social influence and trust were considered in assessing the preparedness of consumers in accessing eHealth services. However, findings in relation to psychological factors and the necessity factor reflect the unpreparedness of participants towards using eHealth services and the advanced healthcare system in Neom, not from a skills perspective, but from a behavioural perspective. Therefore there is a need to streamline the change process by slowly integrating eHealth into the community and creating awareness to implement the change effectively and efficiently.

Thus this study has identified various influencing factors on consumers’ attitudes in using eHealth applications and their implications for developing the Neom project. There are few limitations observed in this study. First, this study adopted a single-method survey instrument for data collection. Using a mixed methods approach by adopting other data collection methods, such as qualitative interviews, can lead to collection of quality data, especially investigating the behavioural factors in detail, which could have improved the scope of influencing factors and their analysis from multiple perspectives. In addition, the small sample achieved in this study makes it difficult to generalise the results. With a lack of existing literature in the context of evolving influencing factors in the use of eHealth in Saudi Arabia, the findings and limitations in this study can guide future research. Areas such as healthcare management; integration of AI into healthcare; health information management; and ethical, procedural and regulatory issues in using advanced healthcare technologies in the context of Neom could be ideas for future research. In addition, findings in this study can have practical implications, as it can support decision makers in better analyses of consumers of eHealth and better prepare them for advanced innovative and predictive eHealth technologies.

## Data Availability

Data availability statement on request.
